# Towards an effective refactoring taxonomy for sustainable software systems

**DOI:** 10.1371/journal.pone.0336296

**Published:** 2026-01-13

**Authors:** Abdullah Almogahed, Hairulnizam Mahdin, Said Badreddine, Mazni Omar, Abdulwadood Alawadhi, Samera Obaid Barraood, Manal Othman, Shazlyn Milleana Shaharudin, And Siti Salwani Yaacob

**Affiliations:** 1 Faculty of Computer Science and Information Technology, Universiti Tun Hussein Onn Malaysia, Parit Raja, Johor, Malaysia; 2 Network and Security Department, Faculty of Computer Information Science, Higher Colleges of Technology, Al Ain, Abu Dhabi, UAE; 3 School of Computing, Universiti Utara Malaysia, Sintok, Malaysia; 4 Department of Computer Science, College of Computers and Information Technology, Hadhramout University, Hadhramout, Yemen; 5 Computer Science Department, Faculty of Applied Sciences, Taiz University, Taiz, Yemen; 6 Department of Mathematics, Faculty of Science and Mathematics, Universiti Pendidikan Sultan Idris, Tanjung Peral, Perak, Malaysia; 7 Faculty of Computing, Universiti Malaysia Pahang Al-Sultan Abdullah, Pekan, Pahang, Malaysia; West Pomeranian University of Technology, POLAND

## Abstract

Software refactoring is pivotal in improving software quality and ensuring complex software systems’ long-term viability and sustainability. However, developers still face challenges in understanding how refactoring approaches influence software quality, as existing classifications largely overlook sustainability aspects and provide limited guidance for long-term software evolution. This gap creates difficulties for developers in selecting appropriate refactoring approaches supporting quality improvement and sustainable maintenance. Therefore, this study addresses these challenges by proposing a novel refactoring taxonomy based on estimated external quality attributes (EEQAs). The proposed taxonomy was developed through three key phases: exploratory study, experimental study, and multi-case analysis. The exploratory study identified the most used refactoring approaches in practice, the EEQAs (effectiveness, extendibility, flexibility, functionality, reusability, and understandability), and five case studies. In the experimental study, 41 experiments across the case studies were conducted to assess the influence of refactoring approaches on EEQAs. Then, a multi-case analysis was performed to examine these effects across all case studies. The proposed refactoring taxonomy categorized the refactoring approaches into positive, negative, and ineffective groups by using their aggregate impact on the EEQAs. The proposed taxonomy puts software sustainability practices at the forefront, prioritizing EEQAs: effectiveness, extendibility, flexibility, functionality, reusability, and understandability. It offers developers a systematic approach to improving software quality, aligning with user expectations, supporting future enhancements, and facilitating system maintenance. By prioritizing these attributes in refactoring practices, developers can build sustainable, robust, and maintainable software systems that deliver long-term value to stakeholders, effectively manage the complexity of software systems, and maintain high code quality standards over time.

## 1. Introduction

Software systems are undergoing regular modifications to meet new requirements, correct existing flaws, and enhance current functionality [1]. Sustainable software engineering consists of developing software capable of dealing with current requirements and having the overall technological ability to meet future requirements [2]. Becker et al. [3] contend that software sustainability includes five interconnected dimensions: individual, social, economic, environmental, and technical. Individual sustainability is acting on the side of human impacts, while social sustainability is concerned with the effects on communities and society [4]. Economic sustainability addresses the ability to ensure long-term financial stability [4], whereas technical sustainability means a system’s longevity, ease of maintenance, and adjustments [3,4].

Software sustainability refers to software available today and will continue to be improved and supported in the future [5]. This implies that sustainability is concerned with software availability, extensibility, and maintainability as the fundamental pillars of sustainability [5]. Venters et al. [5] linked software sustainability to non-functional requirements known as software quality attributes, proposing that external quality attributes such as flexibility, maintainability, usability, extensibility, effectiveness, and reusability can be used to estimate software sustainability. In this context, adopting high-quality source code is the ultimate way through which software evolution can be ensured as sustainable [1,6,7]. Indeed, several experimental studies feature evidence documenting that maintenance and evolution are hampered by low-quality code [1,6,7]. Thus, specific techniques were recommended to help programmers achieve high code quality using software refactoring [1,8].

### 1.1 Software refactoring

Software refactoring is a vital software engineering technique used to ensure the sustainability and maintainability of complex software systems [1,8–10]. This technique represents the systematic approach aimed at rebuilding and refactoring the existing source code, which is principally framed around the internal improvements throughout the code with a focus on the preservation of the program’s functionality [9–11].

Refactoring includes polishing the code to make it more readable, maintainable, and efficient [12].

This practice has been identified to have tremendous significance as software systems have now become highly intricate and dynamic [13]. In the face of the ever-changing and evolving nature of IT and the end-user dynamics, refactoring in software development has never been more critical [14]. This way, refactoring guarantees timelessness, smooth-sailing updates, and modifications to the program’s future and preserves its intended functions and outputs without compromising any part of it [15].

Over the last few years, refactoring has become a routine practice that aims to increase software quality and is the key contributor to software systems’ continuous improvement and evolution [16]. Fowler et al. [9] were the first ones to define 68 refactoring approaches, and later on, they divided them systematically into six different groups. Each particular refactoring approach belongs to one of the corresponding categories that state exactly how and where it can be implemented on given code components, for example, attributes, methods, or classes [9,10].

### 1.2 Refactoring challenges

Identifying how refactoring approaches impact the quality of the software is still a significant challenge [17–24]. Several studies were conducted on the empirical basis of the influence of refactoring approaches on software quality.

These studies aimed not only to find out whether or not using these approaches made changes in the quality characteristics of software but also to check whether or not improving the quality characteristics of software can be realized through the application of these approaches.

Based on the in-depth analysis of the literature concerning refactoring, various researchers have reached various conclusions in terms of the quality of software. Some of the studies, such as [25–28], and [29], showed the positive impact of refactoring on increasing the software quality. A contrary view, however, was defended by [30] and [31], stating that these approaches had detrimental effects on the software quality. Moreover, other studies [32,33], and [34] imply that refactoring approaches have no noticeable impact on software quality.

Finally, numerous studies, among others, performed by [35,36], and [37] have yet to reach a definite conclusion; thus, the ambiguity is on whether refactoring significantly affects the quality of software products. These inconsistent outcomes raise numerous issues for developers aiming to improve the established software by the refactoring approaches [11,15,23,38]. The studies [15,38], and [39] called attention to the struggles that programmers face when comparing the pros and cons of each refactoring approach [40].

At this point, the situation becomes even more intricate in the face of the contradicting results of the different approaches. Furthermore, in works [11,23], and [40], research was conducted to show the hardships programmers experience in deciding the best fitting refactoring approaches among a list to solve particular design problems. In addition, Nyamawe et al. [41] and Almogahed et al. [42] noted that developers still need help deciding on the best refactoring approaches.

The discussion currently going on among software researchers is centered around whether a system’s quality attributes are influenced by different refactoring approaches [11,15,23,28]. Consequently, developers are faced with the challenges of selecting an appropriate refactoring approach to improve code quality, as specified by addressing design flaws [43–48]. Several studies [11,18,19,23] have pointed out a need for research into the influence of refactoring approaches on estimated external quality attributes (EEQAs). These dimensions, including effectiveness, extendibility, flexibility, functionality, reusability, and understandability, are the attributes that have not yet been thoroughly investigated in the context of refactoring impacts [11,18,19,23]. These EEQAs can measure software sustainability, where improving these attributes leads to enhancing the sustainability of the software system [5]. This research gap could provide a direction where more empirical studies and thorough research could be carried out to show how refactoring affects real-world quality aspects.

### 1.3 Study contributions

To the best of our knowledge, there is still a lack of taxonomy that can classify the refactoring approaches based on the EEQAs. Accordingly, this study addresses the research question: How can commonly use refactoring approaches be systematically classified into an effective taxonomy, based on their impact on EEQAs, to support sustainable software development?. Consequently, the main contribution of this study is to propose a novel refactoring taxonomy that classifies the most commonly used refactoring approaches in practice based on important EEQAs, such as effectiveness, extendibility, flexibility, functionality, reusability, and understandability. The contributions of this study are summarized as follows:

- Identifying ten refactoring approaches commonly used in practices and the EEQAs.
- Investigating the effects of each refactoring approach on the EEQAs in five case studies.
- Proposing a novel taxonomy to classify the refactoring approaches based on the EEQAs.

This proposed refactoring taxonomy provides a systematic roadmap for experts to lift software quality to a higher level via the proper selection and goal-oriented implementation of fitting refactoring approaches. The proposed taxonomy will help software practitioners have a more robust knowledge of how the relief amount impacts the variety of quality attributes. As a result, the proposed refactoring taxonomy enables practitioners to choose approaches that comply with their design goals, enhancing the software’s quality within the particular context. In this case, the proposed refactoring taxonomy dramatically simplifies the workflow for developers, who would otherwise apply much effort and time to choose the most effective refactoring approach. This is especially important given the often-challenging nature of reconciling conflicts and trade-offs among these approaches. The proposed taxonomy is founded upon empirical evidence and offers a potentially fruitful approach to reducing software maintenance costs by mitigating associated risks. Moreover, by enabling programmers to choose the most effective refactoring approaches to enhance EEQAs, the software systems become more sustainable for future improvements and evolution.

This paper consists of five sections. The related work is elaborated upon in Section 2. The methodology utilized to propose the refactoring taxonomy is detailed in Section 3. The findings are discussed in Section 4, and threats to validity are discussed in Section 5. The conclusion is presented in Section 6. The limitations and future directions are presented in Section 7.

## 2. Related work

This section discusses in detail the previous studies on the influence of refactoring approaches on the quality of software systems and the previously proposed categorizations of refactoring approaches based on the quality attributes.

### 2.1 Effect of refactoring on software quality

A study by [28] analyzed the connection between several refactoring approaches and various inheritance metrics using three Eclipse user interface application versions. They analyzed the likelihood of refactoring at different inheritance depths. The findings indicated that most refactoring was done at a second-level depth of inheritance. As the inheritance depth grew, the code’s complexity also increased. Palomba et al. [29] examined refactoring approaches in three open-source Java projects, finding that classes with many bug fixes leaned towards refactoring for comprehensibility and maintainability, while those with numerous new features focused on cohesion. The study emphasized the necessity of models guiding developers on suitable refactoring approaches to enhance quality based on specific contexts.

Using three open-source projects, a study [32] examined the link between refactoring approaches and quality metrics. According to their findings, refactoring frequently failed to improve quality, as there was no evident correlation. Shahjahan et al. [49] experimented to evaluate the influence of ten refactoring approaches on resource utilization, time behavior, and changeability. They also examined internal metrics such as class coupling, code lines, depth of inheritance, and cyclomatic complexity. The outcomes demonstrated an improvement in software quality.

Additionally, a study by [50] investigated the effect of some refactoring approaches on complexity. Their observation revealed a reduced complexity, suggesting an enhancement in the project’s quality. Consequently, they showed a decrease in maintenance expenses. Khrishe and Alshayeb [51] undertook an empirical analysis to determine if the sequence in which refactoring approaches are applied influences code quality. They conducted six experiments on a custom-made C# class with nine methods using three distinct refactoring methods in varying sequences. Their findings highlighted that the sequence in which refactoring is applied impacts code quality. A study [52] conducted an experiment concentrating on system architecture issues, specifically anti-patterns or bad smells. They used refactoring approaches to alleviate these issues and assessed the impact on various quality metrics. The outcomes of this investigation generally indicated that using refactoring to rectify architectural problems enhanced the overall quality of the projects under scrutiny.

Moreover, Kádár et al. [53] introduced a manually validated dataset from seven projects to analyze the impact of refactoring on code metrics. Their analysis revealed that lower maintainability often prompted refactoring, which beneficially reduced code complexity, coupling, and size. Kaur and Singh [54] investigated the enhancement of code maintainability through various refactoring approaches. While metrics indicated an improvement in maintainability post-refactoring, the study broadly assessed the refactoring without delving into specific approaches.

A study by [55] examined the influence of three refactoring approaches on external quality attributes using UML class diagrams in several projects. They found that these approaches enhanced the quality attributes. A study [56] analyzed the influence of several refactoring approaches on design attributes across various projects’ version histories. The results revealed that 65% of the attributes improved post-refactoring, while 35% saw no change. A study [57] investigated the effect of clone refactoring on various software attributes through the ANT project. Their findings showed enhancements in complexity, coupling, and size characteristics and decreased test code size. A study [58] examined the effect of five refactoring approaches on the security metrics across several software projects. Their research showed that these approaches improved the protection of the evaluated projects. A study [59] investigated the influence of four specific refactoring approaches on design metrics. The findings indicated varied impacts of these approaches on the metrics under study.

The results obtained from the previous studies confirm that the refactoring approach has different effects (not change, improvement, harmful) on software quality attributes. In addition, further research is needed to investigate the impact of refactoring approaches on EEQAs in terms of functionality, reusability, extendibility, flexibility, and understandability [11,18,19,23]. To bridge this knowledge gap, this study investigated the impact of various refactoring approaches on the EEQAs: effectiveness, extendibility, flexibility, functionality, reusability, and understandability.

### 2.2 Refactoring approaches categorization

Several studies have tried classifying the refactoring approaches according to software quality attributes. Bois and Mens [60] categorized and examined the influence of four refactoring approaches (Pull Up Method Superclass, Extract Method, Encapsulate Field, and Pull Up Method Subclass) on five internal quality metrics in a Java package case study. The results indicated that the Encapsulate Field, Extract Method, and Pull Up Method Superclass positively impacted the quality metrics, whereas the Pull Up Method Subclass had a negative effect. Bois, Demeyer, and Verelst [61] introduced a categorization to guide the application of refactoring approaches for enhancing class cohesion and coupling. Their study found that several refactoring approaches (Move Method, Extract Class, Replace Method, Extract Method, and Replace Data) improved coupling and cohesion.

In addition, a study by Elish and Alshayeb [35] proposed categorizing five refactoring approaches (Extract Method, Extract Class, Encapsulate Field, Conditional Expression, and Hide Method). These approaches were classified according to their measurable impact on software metrics (cohesion, coupling, size, and complexity) and one external attribute (testing effort). Elish and Alshayeb [62] classified refactoring approaches, focusing on their effect on design attributes, such as inheritance and size, and external attributes, like adaptability and testability. Based on prior research correlations, this categorization linked internal metric changes due to refactoring with external attributes. Nevertheless, the research conducted through small-scale projects could have been more extensive in scope and needed further empirical testing. This necessitates the development of a more comprehensive taxonomy.

In the same context, Alshayeb et al. [63] proposed a kind of refactoring classification in the context of Aspect-Oriented Programming (AOP), decomposing them into internal metrics like inheritance and coupling, and external attributes like reliability and maintainability. Elish and Alshayeb [64] categorized refactoring approaches to patterns and analyzed their effect on a software system’s internal and external metrics, such as inheritance, coupling, adaptability, and reusability. Such grouping allowed the comparison of internal metric analyses resulting from pattern-based refactoring to external attributes.

Through a study conducted by Malhotra and Jain [65], four specified refactoring approaches were evaluated on both internal and external quality factors (for example, complexity, cohesion, maintainability, and reusability). They utilized CK metrics to classify approaches according to their impact on these attributes. Malhotra and Chug [66] classified five refactoring approaches based on their impact on internal design characteristics (inheritance and coupling), understandability, and modifiability quality attributes.

Furthermore, in their recent study, Almogahed et al. [15] introduced a classification framework for ten refactoring approaches. This framework categorizes these approaches based on their impact on internal characteristics such as encapsulation, polymorphism, inheritance, coupling, cohesion, and messaging. The approaches were categorized into three groups: safe, ineffective, and unsafe, providing developers with a systematic framework to select the appropriate refactoring approach for targeted enhancements in quality attributes. The study, however, was limited to internal quality attributes. Furthermore, Almogahed et al. [38] introduced a model for classifying various refactoring approaches according to their influence on internal attributes. The refactoring approaches have been categorized into three distinct groups: red, green, and yellow refactoring approaches. This model exclusively prioritized internal qualities.

In addition, recent work [94] has also explored the use of artificial intelligence to support sustainable refactoring. For instance, a study proposed a multi-classification refactoring framework using Hopfield Neural Networks (HNN) to classify refactoring strategies according to their impact on external quality attributes. The framework was developed through case studies, experiments, and classification processes, and it categorizes refactoring strategies into positive, negative, and ineffective groups.

However, these categorizations need to be improved. They are not sufficiently comprehensive, as many refactoring approaches and quality attributes are not covered. There needs to be evidence from the industry regarding the selection of refactoring approaches. In most studies, the refactoring approaches included were chosen subjectively by the researchers or based on a literature review analysis. They are limited to a small set of external quality attributes, and EEQAs, such as effectiveness, extendibility, flexibility, functionality, reusability, and understandability, still need to be addressed. Therefore, to address these gaps, this study proposed a refactoring taxonomy for classifying the most commonly used refactoring approaches among practitioners based on the EEQAs.

## 3. Methodology

**Fig 1:**
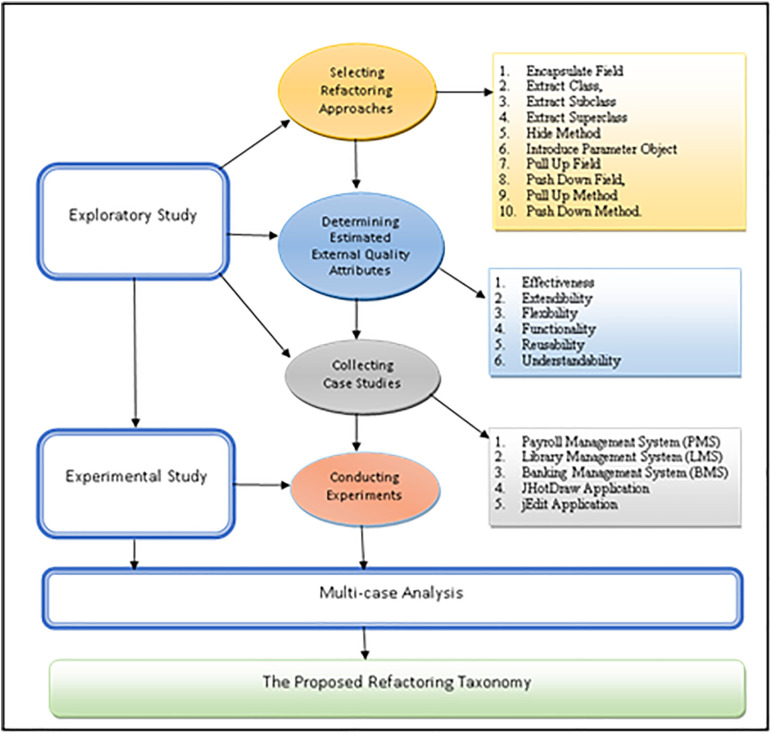
Methodology for developing the proposed refactoring taxonomy.

This section outlines the methodology used to create the refactoring taxonomy depicted in Fig 1. The proposed refactoring taxonomy was formulated through a sequential process consisting of three distinct phases: an initial exploratory study, followed by an experimental study, and finally, a comprehensive multi-case analysis. The subsequent sections offer thorough explanations of each phase.

### 3.1 The exploratory study

In this phase, we have identified the most commonly employed refactoring approaches, EEQAs, and case studies.

#### 3.1.1 Selecting the refactoring approaches.

Fowler et al. [9,10] presented a thorough compilation of 68 unique refactoring approaches, classified into six separate categories, specifically emphasizing object-oriented programming. This research meticulously examined the existing literature to identify the prevalent and widely adopted refactoring approaches. In addition, an array of systematic literature reviews [18–20,67–71] have documented the frequently employed refactoring approaches in practices and academic research. Concurrently, a study [72] scrutinized the most commonly used refactoring approaches by software engineers at Microsoft.

A study [73] enlisted 13 refactoring approaches recognized by ex-industry developers as the most probable candidates for application, representing a comprehensive array of refactorings. Ouni et al. [74] divulged the prevalent refactoring approaches in practical software development. Moreover, Almogahed and Mazni [75] conducted an exploratory study among software practitioners to determine the most frequently employed refactoring approaches in current practice. Ongoing research [11,15,23,38] has unveiled the most widely applied refactoring approaches in practical scenarios. Consequently, guided by the insights gained from the literature review and exploratory analysis, this study identified the ten most frequently employed refactoring approaches.

The selected refactoring approaches are Encapsulate Field(EF) [9–11,18–20], Extract Class (EC) [9–11,18–20], Extract Subclass (ESb) [9,10,18–20], Extract Superclass (ESP) [9–11,18–20], Hide Method (HM) [9–11,18–20], Introduce Parameter Object (IPO) [9,10,18–20], Pull Up Field (PUF)) [9,10,18–20], Push Down Field (PDF) [9,10,18–20], Pull Up Method (PUM) [9,10,18–20] and Push Down Method (PDM) [9,10,18–20].

#### 3.1.2 Identifying estimated external quality attributes (EEQAS).

Numerous quality models have been developed to assess the quality attributes of software products, including the Dromey model, McCall model, Boehm model, FURPS Model, ISO/IEC 9126, ISO/IEC 25010, and quality model for object-oriented design (QMOOD) [76]. For the objectives of this research, a high-quality model is essential for evaluating the quality of software systems and quantifying the impact of refactoring approaches on the quality attributes. The chosen model should have quantitative evaluations of the estimated external quality attributes to achieve this objective. Therefore, the QMOOD model [77] emerges as the most suitable choice for this study in this particular setting.

Our study adopted the QMOOD model based on its synchronicity with Java-based object-oriented systems, which are significant topics of discussion in the literature [11,23]. The model covers all the features of the object-oriented system internally [15,38]. Furthermore, QMOOD was selected to collect the external quality data from the software before and after refactoring approaches were used. The strength of QMODE, which allows software quality analysis on different sides, is a crucial advantage [78,79]. It includes six vital estimated external quality attributes (EEQAs) (understandability, functionality, effectiveness, flexibility, extendibility, and reusability), which are the critical qualities of the software that address the fundamental aspects of a software program. These attributes are precisely suitable for evaluating the desired functionality of object-oriented systems [77,80].

This study will focus on EEQAs. The mathematical equations for calculating EEQAs using software metrics are demonstrated in Table 1. According to Bansiya and Davis [77], the equations entail scaling up metrics to estimate the impact of internal metrics values on external quality attributes. An empirical study of the evolving software systems fixed these weights, including positive (+0.5, + 1) and negative (−0.5, −1) values. The credited weight coefficients lead to the positive effect of the estimated external quality attribute, whereas they do the opposite when the weight coefficients are negative.

**Table 1:** The estimated external quality attributes [77].

Quality attribute	Mathematical Formulas
Total Quality Index	∑ (Reusability + Flexibility + Effectiveness + Extendibility +Understandability + Functionality)
Reusability	−0.25* DCC + 0.25*CAM + 0.5* CIS + 0.5*DSC
Flexibility	0.25 * DAM – 0.25 * DCC + 0.5 * MOA + 0.5 * NOP
Effectiveness	0.2 *ANA + 0.2 * DAM + 0.2 * MOA + 0.2 *MFA + 0.2 * NOP
Extendibility	0.5 * ANA −0.5 * DCC + 0.5 * MFA + 0.5 * NOP
Functionality	0.12* CAM + 0.22*NOP + 0.22*CIS + 0.22*DSC + 0.22*NOH
Understandability	−0.33*ANA + 0.33*DAM – 0.33*DCC + 0.33*CAM −0.33*NOP –0.33 * NOM – 0.33 * DSC

DCC: Direct Class Coupling, CAM: Cohesion Among Methods in a Class, CIS: Class Interface Size, DSC: Design Size in Classes, DAM: Data Access Metric, MOA: Measure of Aggregation, NOP: Number of Polymorphic Methods, ANA: Average Number on Ancestors, MFA: Measure of Functional Abstraction, NOH: Number of Hierarchies, NOM: Number of Methods.

The quality attribute is assessed based on observed changes in weight value across releases of sequential software systems, where increased weight values signify an increase in quality and decreased values indicate the deterioration of the quality attributable to the quality attribute.

The six EEQAs included in this study are defined as follows [77]:

- Reusability: This attribute evaluates the software module’s compatibility with various software systems.
- Flexibility: This attribute indicates how easily a software module can be modified and adjusted to function in settings outside of the scope of its original design.
- Effectiveness: This attribute evaluates the design’s capability to achieve the intended functionality and behavior using object-oriented principles.
- Extendibility: This attribute shows how easy it is to incorporate new requirements into the current design, enabling adjustments to improve the functional capability of the software component.
- Understandability: This attribute deals with the design properties leading to comprehending software by everyone.
- Functionality: This attribute refers to the properties of the design class made publicly accessible by their corresponding public interface, determining the services that this class provides.

#### 3.1.3 Collecting the case studies.

This section presents the selected case studies for this study, which were obtained from two separate settings: practical and theoretical. One of the reasons why we chose these case studies is that the developers in these studies vary in their programming levels from beginners to expert veterans and professionals [15,23,62]. Moreover, the case studies were taken with different sizes as the scale was small to medium and large. As refactoring occurs in several domains, accuracy and consistency of the assessment are crucial and provide evidence on whether the refactoring approach is suitable for different quality dimensions in diverse settings and various case study sizes.

These case studies consist of open-source projects accessible through online software repositories such as GitHub and SourceForge, and they were readily available for download without any cost. The choice of these case studies was driven by their extensive utilization in previous refactoring studies [11,18–20,23,38,81]. Table 2 presents a concise overview of the examined case studies.

**Table 2:** Statistical analysis of examined case studies.

Case Study	Number of classes	Lines of code (LOC)	Size	Environment	Programming language
Payroll Management System (PMS)	12	1,263	Small	Academic	Java
Library Management System (LMS)	19	1,570	Small	Academic	Java
Bank Management System (BMS)	34	3,689	Small	Real-World	Java
jHotDraw	250	14,866	Medium	Real-World	Java
jEdit	1,153	122,699	Large	Real-World	Java
**Total**	**1,468**	**144,087**			

The five case studies are described as follows.

- Payroll Management System (PMS): The PMS is a small-scale application comprising 12 classes. It has been constructed with the primary objective of automating employee payroll management and enhancing organizational management processes. The PMS is accessible online and can be downloaded via this link [82].
- Library Management System (LMS): A small-scale application with 19 classes developed to facilitate library management. It offers various features for librarians to maintain records of available and borrowed books, aiding in organizing and managing library operations. LMS is accessible online at [83].
- Banking Management System (BMS): A small-scale application comprising 34 classes. It serves the purpose of managing essential data required for calculating monthly account statements for bank customers. BMS offers various customer services and is designed to meet the process requirements of a bank, ultimately enhancing the bank’s productivity. The system is available on the GitHub repository and can be accessed and downloaded online from this link [84].
- jHotDraw Application: A moderately-sized open-source project with approximately 250 classes. This two-dimensional graphics framework, developed in Java, is a foundation for structured drawing editors. It facilitates the creation of GUI-based editors with tool palettes and multiple views. Moreover, it allows customization using inheritance and component combinations. From a software engineering perspective, jHotDraw is significant as one of the early projects intentionally designed for reusability and labeled as a framework. jHotDraw 5.2 is available online at this link [85].
- jEdit Application: It is a large-scale open-source project boasting 1,153 classes written in Java. This cross-platform text editor is tailored for programmers and functions across various operating systems that support Java. jEdit offers advanced features for over 130 programming languages. It is essential to clarify that jEdit is a text editor, not a word processor, and it excels in manipulating source code, markup text, and other text files. Focusing on programmers, it provides project management tools and works with other programming tools. The most recent stable version, jEdit 5.5.0, is available from the Sourceforge repository and can be accessed here [86].

### 3.2 Experimental study

**Fig 2:**
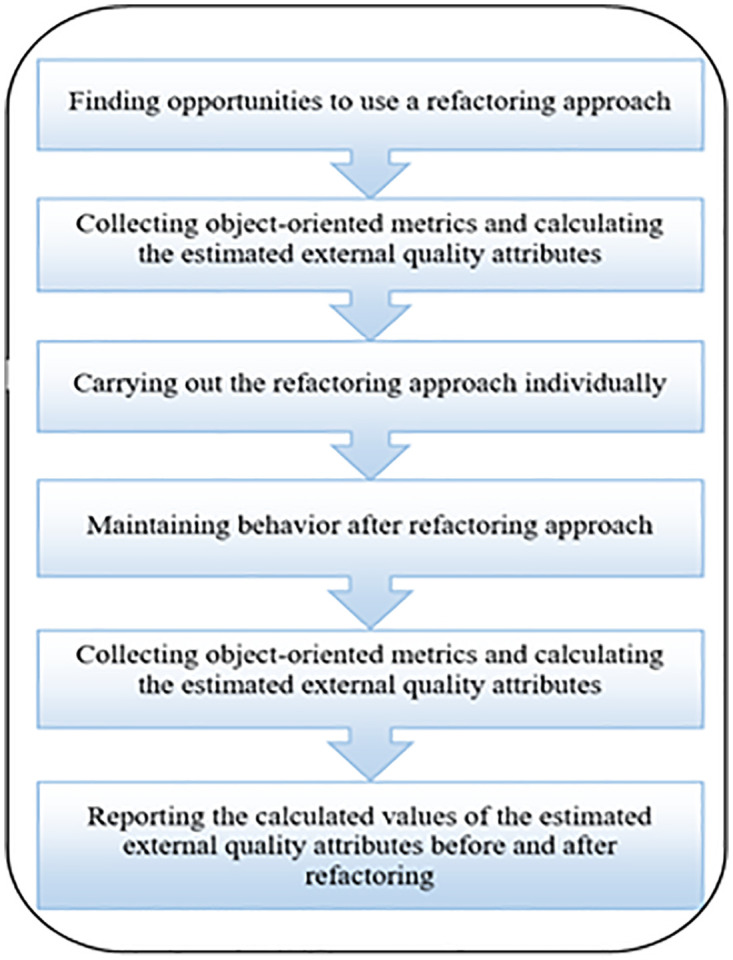
Experimental procedure.

The experiments have been conducted during this phase. The experimental procedures implemented to investigate the impact of each refactoring approach on the EEQAs for each case study are outlined in Fig 2.

The experiments were conducted using a series of steps, which are outlined as follows:

#### 3.2.1 Figuring out opportunities for applying a refactoring approach.

This step involved analyzing the classes that comprise a software project to identify possible parts requiring refactoring to enhance the overall design quality. Fowler provided examples in his book to guide readers through the process of utilizing the refactoring approaches [9,10].

By the time this step was over, the potential classes requiring refactoring had been detected, and the refactoring approach associated with those classes had been chosen. JDeodorant Tool [87] helped identify the opportunities to use one refactoring approach (Extract Class). However, it was checked manually to guarantee accuracy. The opportunities for using the other refactoring approaches were identified manually.

#### 3.2.2 Collecting metrics and calculating the EEQAs before implementing a refactoring approach.

The software metrics (NOM, ANA, NOP, MFA, MOA, NOH, DCC, CIS DSC, DAM, and CAM) were employed to calculate the values of the EEQAs (effectiveness, extendibility, flexibility, reusability, functionality, and understandability). Table 1 in Section 3.1.2 provides a detailed explanation of the mathematical equations used to calculate the EEQAs. The metrics were gathered in an automated manner using the Eclipse Metrics plugin 1.3.8 [88]. Furthermore, the EEQAs were calculated utilizing a Microsoft Excel spreadsheet due to the absence of automatic inclusion tools.

#### 3.2.3 Implementing the chosen refactoring approach on an individual basis.

The chosen refactoring approaches were implemented individually to assess their impact on the EEQAs. Fowler explained the operational principles behind each refactoring approach [9,10]. The implementation of refactoring approaches can be carried out either manually or using tools for specific refactoring approaches.

JDeodorant tool performed the Extract Class refactoring approach, while the Encapsulate Field refactoring approach used Eclipse [89]. The rest of the refactoring approaches were executed fully manually, as no tools are available to implement them automatically. The manual verification of these refactoring techniques has been conducted to ensure their adherence to the mechanics proposed by Fowler [9,10], as the current refactoring tools are prone to errors.

Consequently, using these tools may result in inaccurately refactored parts of code [18,20]. The remaining refactoring approaches were executed manually, mirroring recent investigations [11,15,23,38], which were founded on the principles of operation outlined by Fowler [9,10].

#### 3.2.4 Ensure that behavior is preserved after implementing each refactoring approach.

Behavior preservation of the system’s components refers to the requirement that the outputs generated by the system remain unchanged after applying the refactoring approach, as they were before the refactoring was performed [11,15,18,19,23,38]. Similar to these previous studies [11,15,23,38], regression testing was employed to achieve the preservation of the systems’ functionality.

In this study, regression testing was carried out by compiling and executing the source code both before and after applying each refactoring step, and then analyzing the outcomes to determine whether any differences occurred. If the outputs were identical, the refactoring was considered correct and retained; if discrepancies were observed, the refactoring was rejected.

To further strengthen the verification, we manually tested the main functional use cases of the system after each refactoring step. This manual validation ensured that core system behavior, input–output consistency, and expected user interactions were preserved. Although automated testing frameworks such as JUnit are commonly used in Java-based projects, in this work, the combination of compilation checks and manual functional testing provided sufficient coverage of the system’s main requirements and gave us confidence that no functional behavior was altered as a result of the applied refactorings.

#### 3.2.5 Gathering metrics and computing the EEQAs after implementing a refactoring approach.

After executing the chosen refactoring approach, the metrics were gathered to calculate the pertinent EEQAs. This step was performed precisely as outlined in the aforementioned second step. Finally, report the calculated values of the EEQAs before and after implementing the refactoring approach and preserve the modified source code.

### 3.3 Multi-case analysis for classification refactoring approaches

When determining the general mechanisms underlying complex phenomena or systems, the multi-case analysis is a practical and effective strategy [90,91]. When researchers use this strategy, they can better understand the theoretical constructs underlying new phenomena or systems. Within this study’s scope, the multi-case analysis’s primary objective is to categorize the refactoring approaches based on their effect on the EEQAs.

A comprehensive analysis was conducted on 41 experiments using all collected case studies. The measuring data of the EEQAs before and after refactoring approaches implementations in all experiments were consolidated into a unified data pool. Here, the word pool represents data aggregation from all the experiments before and after the refactoring approach. This aggregated data is treated as a single unit for a multi-case analysis. Metrics and computed values of the EEQAs before and after the refactoring of the five case studies are included in the data. To get to the level of generalizability, cross-case analysis was used to analyze the data and identify and contrast the information [92]. Hence, the process of identifying common and distinct features from the classified cases [93].

Each refactoring approach is classified separately using a common practice design method based on how it affects the EEQAs. By application of this method, the implications of every single refactoring approach on the EEQAs in each case study in the pool were found, compared, and analyzed, noting the frequency of the appearance of each effect in every experiment. Then, the most frequently seen effect was classified and selected for the proposed refactoring taxonomy.

## 4. Results and discussion

This section presents, analyzes, and discusses the findings of the experiments for the case studies in the five cases described in this experimental study. The procedure selected for the investigation was to select ten refactoring approaches during the investigation of the study as they were the most used currently in practice. It was based on the surveys and extensive literature reviews of the existing approaches.

Forty-one refactoring experiments were conducted independently in the five case studies (10 in jEdit, 10 in jHotDraw, 9 in PMS, 6 in LMS, and 6 in BMS), with one experiment for each refactoring approach in each case study. In other words, each refactoring approach was applied independently in each experiment. Each refactoring approach has been investigated in a separate version of the software project in each case study. For example, jEdit employed ten refactoring approaches. As a result, there are ten independent versions of jEdit, with each refactoring approach used in its version.

The experiments began with an in-depth analysis of the source code’s structure (packages, classes, interfaces, methods, and variables) to identify potential opportunities for each refactoring approach to be used. Each opportunity to use each refactoring approach was investigated separately in each case study. The ten refactoring approaches were carried out 847 times throughout the five case studies based on the opportunities identified. Table 3 details the use of various refactoring approaches across the case studies. It includes information on the purpose and opportunities identified, the number of opportunities in each case study, and the method of identifying opportunities (either fully manual or automated with the assistance of the JDeodorant Tool and manual verification).

**Table 3:** The opportunities for using each refactoring approach through the case studies.

Refactoring Approaches	Purpose and Opportunity	Number of Opportunities	Method
Extract Class (EC)	When there is a long class that does work that is supposed to be done by two, such a class has a lot of methods and data. The class is too big and difficult to understand. It needs to be split. The opportunity to use the EC creates a new class and moves the pertinent fields and methods from the source class to the extracted class.	The total was 102. 3 in LMS, 13 in jHotDraw, 2 in PMS, 77 in jEdit, and 7 in BMS.	JDeodorant Tool
Extract Subclass (ESb)	The opportunity for use of the ESb is the revelation that a class has actions used for some class instances and not for others. In other words, the class contains methods and fields that are occasionally useful. This refactoring approach transfers all fields and methods related to a completely separate subclass.	The total was 40. 2 in LMS, 29 in jEdit, 8 in jHotDraw,1 in PMS, and 0 in BMS.	Manual
Extract Superclass (ESP)	Duplicate code is considered one of the bad things in a system. One type of code is two classes with similar methods and fields. The opportunity for using the ESP is to remove this type of duplicate code by creating a superclass and moving the common methods and fields to the superclass.	The total was 21. 4 in LMS, 4 in BMS, 3 in jHotDraw, 10 in jEdit, and 0 in PMS.	Manual
Encapsulate Field (EF)	One of the main principles of object orientation is the encapsulation or hiding of data. This means it should always keep the data private. If data is public, other objects can change data values and access them without knowing the owner. Therefore, the opportunity to use the EF is when the field is public. It makes the field private and provides accessors.	The total was 165. 2 in LMS, 104 in jEdit, 39 in jHotDraw, 20 in PMS, and 0 in BMS.	Manual
Introduce Parameter Object (IPO)	When a group of parameters go together naturally, they should be replaced with an object using the IPO. A specific group of common parameters may be used by multiple methods, whether in one class or multiple classes. This group of classes is a data clump, and an object that carries this data can be replaced. These parameters should be turned into objects to group the data.	The total was 27. 1 in LMS, 8 in jHotDraw, 2 in PMS, 14 in jEdit, and 2 in BMS.	Manual
Hide Method (HM)	The opportunity to use the HM is when a method is not utilized in other classes or within its class hierarchy. This refactoring leads to a change in decisions regarding method visibility.	The total was 451. 2 in LMS, 230 in jEdit, 23 in PMS, 164 in jHotDraw, and 32 in BMS.	Manual
Pull Up Field (PUF)	When subclasses are independently developed, duplicate features can often be found. In particular, some fields may be duplicates, and these fields may have identical names. This case represents the opportunity to use the PUF. This approach moves the similar field from the subclasses into the superclass. Doing so will reduce the duplication by deleting the data declaration from the subclasses.	The total was 17. One is in BMS, one is in PMS, three is in jHotDraw, 12 is in jEdit, and 0 is in LMS.	Manual
Pull Up Method (PUM)	Subclasses grew and evolved independently, causing the same (or almost identical) methods. It is essential to remove duplicate code. Two exact methods can work perfectly, but alterations to one copy may not be taken to the other. The opportunity to use the PUM is when the methods in the subclasses have the same body. It moves them to the respective superclass.	The total was 7. One is in PMS, two is in jHotDraw, four is in jEdit, 0 is in LMS, and 0 is in BMS.	Manual
Push Down Method (PDM)	The opportunity to use the PDM is when a method is implemented on a superclass and is only pertinent for one subclass or some of its subclasses. This refactoring removes the method from the superclass and puts it on the subclass(es).	The total was 10. 2 in PMS, 4 in jHotDraw, 4 in jEdit, 0 in LMS, and 0 in BMS.	Manual
Push Down Field (PDF)	The opportunity to use the PDF is when a field is only utilized by one subclass or some subclasses. This approach moves the field to the relevant subclass(es).	The total was 7. 3 in BMS, 2 in PMS, 1 in jHotDraw, 1 in jEdit, and 0 in LMS.	Manual

As shown in Table 3, the opportunities for using one refactoring approach (Extract Class) were identified automatically with the assistance of the JDeodorant tool, and its identification was checked manually to ensure that refactoring approaches were correctly identified. The opportunities for the use of other refactoring approaches were identified manually.

In addition, as shown in Table 3, the opportunities for using refactoring approaches in each case study were based on the availability of these opportunities. For example, opportunities for using the Encapsulate Field were identified in four case studies (i.e., LMS, PMS, jHotDraw, and jEdit). In contrast, the BMS case study found no opportunity to use the Encapsulate Field.

The Eclipse Metrics tool 1.3.8 was used to collect all software metrics. All EEQAs were computed using the Microsoft Excel spreadsheet based on the mathematical formulas provided in QMOOD, as shown in Table 1. Table 4 reports the calculated values of the EEQAs before using refactoring approaches in jEdit, jHotDraw, PMS, BMS, and LMS case studies

**Table 4:** The EEQAs values before implementing refactoring approaches across the five case studies.

Case Study	Reusability	Flexibility	Effectiveness	Extendibility	Functionality	Understandability
LMS	18.25	1	3.6842	−3.2895	9.240	−7.06893
BMS	79.818	10.5	11.4648	−18.838	40.713	−82.18716
PMS	36.1965	4.25	3.1834	1.9585	17.274	−21.83233
jHotDraw	825.009	73.008	91.660	−148.866	460.384	−853.446
jEdit	2880.686	308.420	320.578	−500.895	1499.109	−2458.429

Each refactoring approach was performed individually through five case studies based on the mechanics described by Fowler [1,2] to identify its effect on the EEQAs. Two refactoring approaches were performed using the tools. The Eclipse refactoring tool performed one approach (Encapsulate Field), while the JDeodorant tool performed one refactoring approach (Extract Class). Nonetheless, a manual validation was conducted to verify that they had been carried out as the mechanics recommended by Fowler [1,2]. The other refactoring approaches were manually implemented following the recommended guidelines [1,2].

Table 5 presents the computed values of the EEQAs after performing the refactoring approaches in the five case studies, where the total applied indicates the overall frequency of the refactoring approach implemented in the case study. Six refactoring approaches were used in LMS, six in BMS, nine in PMS, ten in jHotDraw, and ten in jEdit based on the opportunities identified in the five case studies.

**Table 5:** The values of EEQAs after implementing refactoring approaches throughout the five case studies.

Case Study	Refactoring Approaches	Total Applied	Reusability	Flexibility	Effectiveness	Extendibility	Functionality	Understandability
LMS	Encapsulate Field	2	17.25	1	3.6842	−3.2895	8.800	−7.06893
	Extract Class	3	35.16675	2.5	4.791	−5.0225	17.020	−18.24504
	Extract Subclass	2	19.25	1.5	4.1334	−3.1665	10.120	−7.15011
	Extract Superclass	4	19.75	1.25	4.0174	−2.9565	10.78	−8.27871
	Introduce Parameter Object	1	19.5	1	3.85	−3.875	9.900	−8.0025
	Hide Method	2	17.25	1	3.6842	−3.2895	8.800	−7.06893
BMS	Extract Class	7	92.05275	13.5	14.4196	−23.951	47.225	−89.06271
	Extract Superclass	4	65.12975	10.25	12.321	−19.1975	35.462	−73.94838
	Introduce Parameter Object	2	82.03225	10.5	12.05	−20.375	42.015	−84.18993
	Pull Up Field	1	79.818	10.25	11.2648	−18.838	40.713	−82.51716
	Push Down Field	3	79.818	10.75	11.6648	−18.838	40.713	−81.85716
	Hide Method	32	63.818	10.75	11.6648	−18.838	33.673	−81.85716
PMS	Encapsulate Field	20	56.3215	4.5	3.3834	1.9585	26.134	−22.64823
	Extract Class	2	49.33225	5.25	3.9572	0.893	23.279	−30.51081
	Extract Subclass	1	40.268	4.5	3.3846	1.9615	19.069	−24.04083
	Introduce Parameter Object	2	41.15925	4.25	3.5572	0.893	19.676	−25.45917
	Pull Up Field	1	36.1965	4.25	3.1834	1.9585	17.274	−21.82323
	Push Down Field	2	36.1965	4.25	3.1834	1.9585	17.274	−21.82323
	Pull Up Method	1	35.22375	4.25	3.1834	1.9585	16.847	−21.12726
	Push Down Method	2	37.6225	4.25	3.1834	1.9585	17.899	−22.91091
	Hide Method	23	24.6965	4.25	3.1834	1.9585	12.214	−21.82323
jHotDraw	Encapsulate Field	39	850.920	73.300	91.894	−148.866	471.782	−860.438
	Extract Class	13	875.91175	77.75825	98.245	−163.904	486.798	−889.51896
	Extract Subclass	8	829.5425	80.75825	96.475	−145.329	467.380	−862.66587
	Extract Superclass	3	826.834	71.50825	91.073	−146.834	461.320	−849.40779
	Introduce Parameter Object	8	823.43125	61.25825	94.0524	−181.3855	466.147	−881.91543
	Pull Up Field	3	827.954	71.508	90.460	−148.866	461.678	−857.148
	Push Down Field	1	825.009	72.75825	91.4602	−148.866	460.384	−853.77567
	Pull Up Method	2	822.707	73.508	92.060	−147.866	459.579	−851.534
	Push Down Method	4	826.038	71.258	91.260	−151.366	460.738	−853.408
	Hide Method	164	743.009	73.008	91.660	−148.866	424.304	−853.446
jEdit	Encapsulate Field	104	2979.109	311.641	323.155	−500.895	1542.392	−2510.379
	Extract Class	77	3148.733	331.730	353.810	−580.436	1635.192	−2643.061
	Extract Subclass	29	2956.912	332.128	339.152	−514.877	1552.238	−2522.878
	Extract Superclass	10	2893.882	308.174	321.187	−499.381	1506.363	−2456.196
	Introduce Parameter Object	14	2902.652	309.358	324.725	−512.903	1511.413	−2480.332
	Pull Up Field	12	2893.001	306.170	319.778	−502.895	1504.860	−2466.594
	Push Down Field	1	2880.686	308.420	320.578	−500.895	1499.109	−2458.429
	Pull Up Method	4	2877.711	308.420	320.578	−500.895	1497.801	−2455.426
	Push Down Method	4	2878.932	307.170	320.178	−502.395	1498.007	−2457.114
	Hide Method	230	2765.686	308.420	320.578	−500.895	1448.509	−2458.429

The green color represents refactoring approaches that improve the EEQA, while the red color represents refactoring approaches that impair the EEQA. Ineffective refactoring approaches are represented by yellow color.

The effect of each refactoring approach on the EEQAs was analyzed based on the compared calculated value of the EEQAs before and after the refactoring approach was performed through the five case studies. How the differences between the original and computed values post-refactoring would be interpreted depended on the QMOOD. In addition, the influence of each refactoring approach was investigated by subtracting the EEQAs measured values before refactoring from the measured values of the EEQAs after refactoring. The refactoring approach increases the related quality attribute if the difference between the two measured values is positive. However, if the difference between the two measured values is negative, the refactoring approach adversely affects the related quality attribute. Finally, if the difference between the two measurements is zero, the refactoring approach does not affect the corresponding quality attribute.

Figs 3–12 illustrate the effect of each refactoring approach on the EEQAs through five case studies.

**Fig 3:**
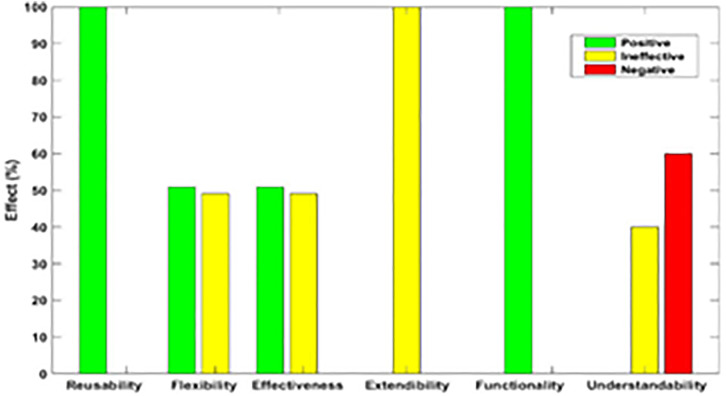
Effects of the Encapsulate Field on the EEQAs.

**Fig 4:**
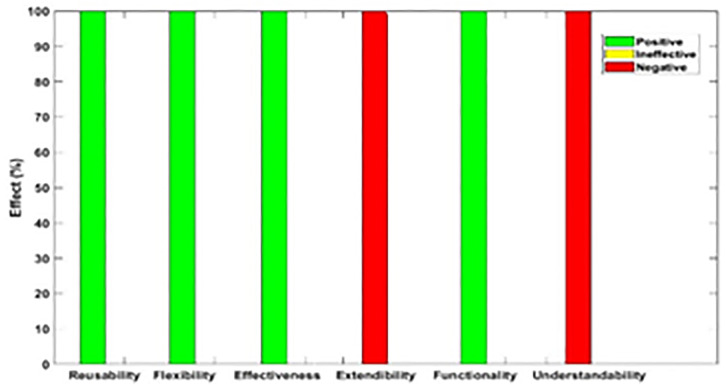
Effects of the Extract Class on the EEQAs.

**Fig 5:**
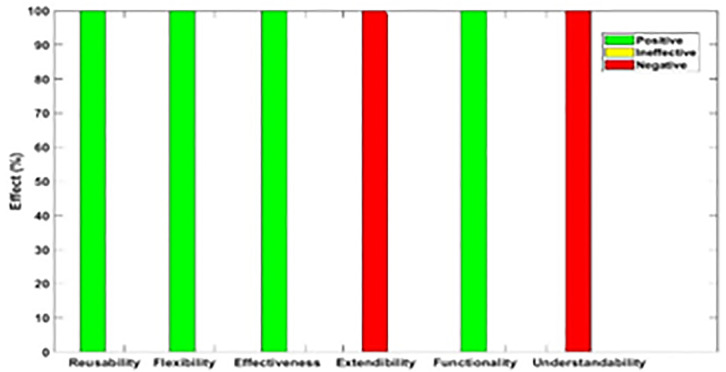
Effects of the Extract Subclass on the EEQAs.

**Fig 6:**
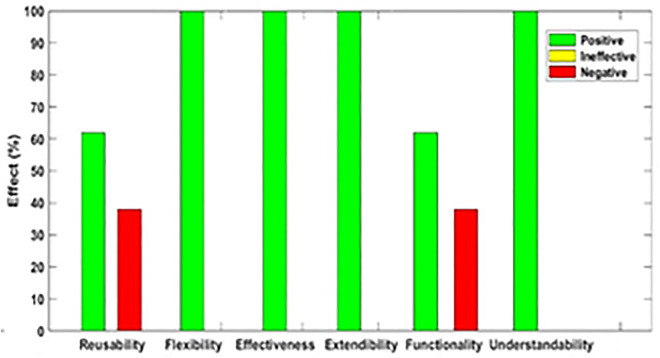
Effects of the Extract Superclass on the EEQAs.

**Fig 7:**
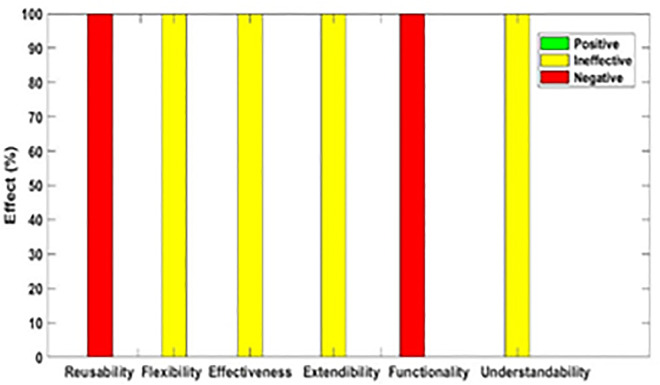
Effects of the Hide Method on the EEQAs.

**Fig 8:**
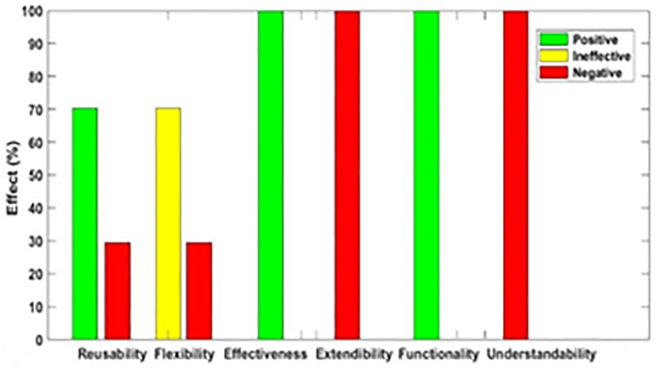
Effects of the Introduce Parameter Object on the EEQAs.

**Fig 9:**
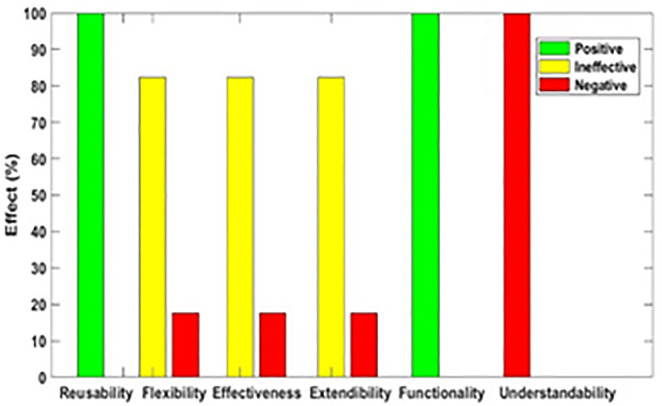
Effects of the Pull Up Field on the EEQAs.

**Fig 10:**
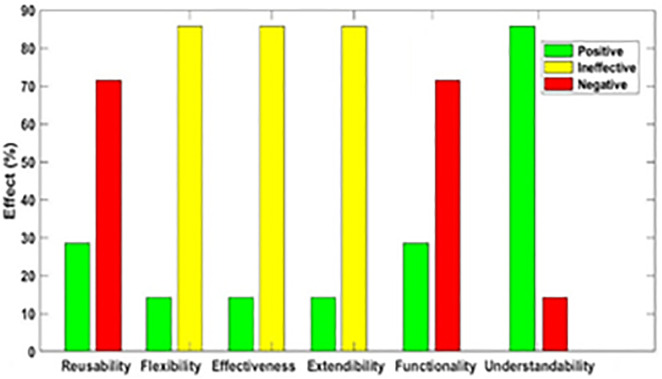
Effects of the Pull Up Method on the EEQAs.

**Fig 11:**
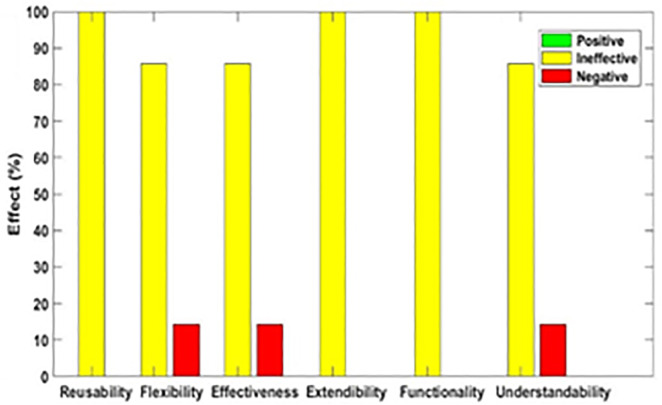
Effects of the Push Dawn Field on the EEQAs.

**Fig 12:**
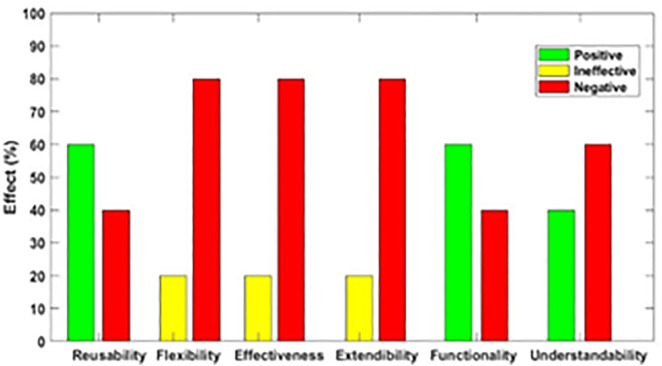
Effects of the Push Dawn Method on the EEQAs.

Following this analysis, the frequency with which every single influence (impair, enhance, or have no change) occurred on the EEQAs was tallied for each refactoring approach. Subsequently, the proportion of each impact was calculated. The highest proportion of effect was classified and selected to be included in the proposed taxonomy. This procedure was repeated for the ten refactoring approaches across the five case studies. The refactoring approaches were categorized into the subsequent three classifications:

- Positive refactoring approach: An EEQA is enhanced by a refactoring approach.
- Negative refactoring approach: An EEQA is compromised by a refactoring approach.
- Ineffective refactoring approach: An EEQA remains unaffected by a refactoring approach.

**Fig 13:**
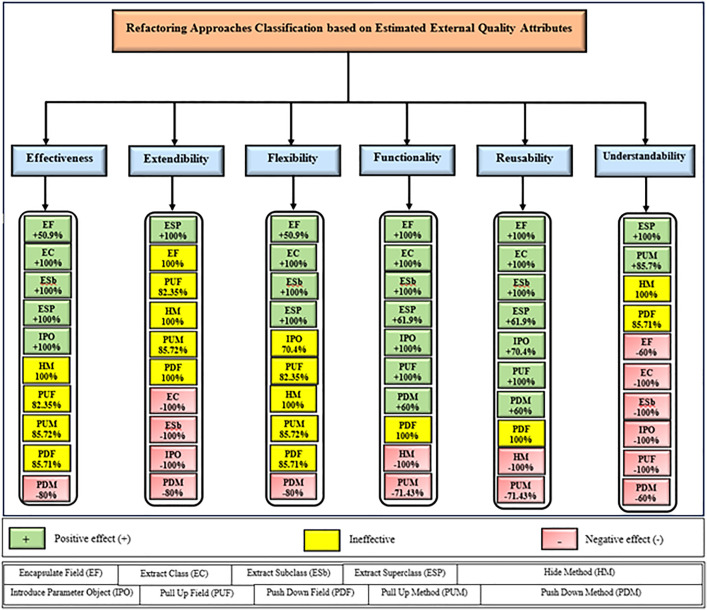
The proposed refactoring taxonomy.

**Fig 14:**
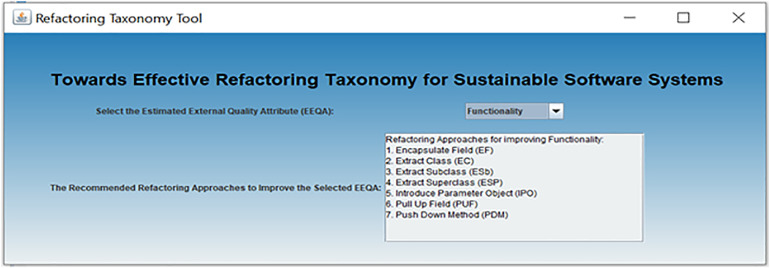
Refactoring taxonomy tool.

Fig 13 presents the proposed taxonomy for classifying the refactoring approaches based on their cumulative effects on the EEQAs. In addition, we have developed a refactoring taxonomy tool in Java to help software practitioners use the proposed taxonomy in a more practical way. This tool helps practitioners select appropriate refactoring approaches that can improve specific EEQAs. Fig 14 shows the developed refactoring taxonomy tool. In this tool, the software practitioners can easily select their prioritized EEQAs from the dropdown list, and the outputs in the text area will be the recommended factoring approaches. Therefore, the software practitioners can choose the most appropriate approaches

The Extract Class (EC) significantly enhanced effectiveness (+100%), flexibility (+100%), functionality (+100%), and reusability (+100%), making it a positive approach for these attributes. EC significantly reduced extendibility (−100%) and understandability (−100%), leading to its classification as a negative approach in these attributes.

The Encapsulate Field (EF) improved effectiveness (+50.9%), flexibility (+50.9%), functionality (+100%), and reusability (+100%); consequently, EF was categorized as a positive approach for these attributes. EF impaired understandability (−60%), which was classified as a negative approach for this attribute. EF did not change extendibility (100%) and was categorized as an ineffective approach for this attribute.

The Introduce Parameter Object (IPO) significantly enhanced effectiveness (+100%), functionality (+100%), and reusability (+70.4%), making it a positive approach for these attributes. IPO negatively impacted extendibility (−100%) and understandability (−100%), leading to its classification as a negative approach for these attributes. IPO did not alter flexibility (70.4%), and it was an ineffective approach for this attribute.

The Hide Method (HM) significantly reduced functionality and reusability by 100%, making it a negative approach in these attributes. HM was deemed ineffective for effectiveness, extendibility, flexibility, and understandability, as it scored 100% in each of these attributes.

The Pull Up Field (PUF) significantly enhanced functionality (+100%) and reusability (+100%); consequently, it was categorized as a positive approach for these attributes. PUF impaired understandability (−100%) and was classified as a negative approach for this attribute. PUF did not modify effectiveness (82.35%), extendibility (82.35%), and flexibility (82.35%), making it an ineffective approach for these attributes.

The Pull Up Method (PUM) improved understandability (+85.7%) and was categorized as a positive approach for this attribute. PUM impaired functionality (−71.43%) and reusability (−71.43%); consequently, it was classified as a negative approach in these attributes. PUM did not modify effectiveness (85.72%), extendibility (85.72%), and flexibility (85.72%), making it an ineffective approach for these attributes.

The Push Dawn Method (PDM) improved reusability (+60%) and flexibility (+60%) and was categorized as a positive approach for these attributes. PDM impaired effectiveness (−80%), extendibility (−80%), flexibility (−80%), and understandability (−60%); consequently, it was categorized as a negative approach for these attributes.

The Extract Subclass (ESb) significantly enhanced effectiveness (+100%), flexibility (+100), functionality (+100), and reusability (+100%), thus being classified as a positive approach for these attributes. ESb showed complete impairment in extensibility (−100%) and understandability (−100%), resulting in its classification as a negative approach in these attributes.

The Extract Superclass (ESP) improved effectiveness (+100%), extendibility (+100%), flexibility (+100%), functionality (+61.9%), reusability (+61.9%), and understandability (+100%), resulting in a positive categorization for these attributes.

The Push Down Field (PDF) did not change effectiveness (85.71%), extendibility (100%), flexibility (85.71%), functionality (+100%), reusability (+100%), and understandability (+85.71%). Therefore, it was classified as ineffective for these attributes.

The proposed refactoring taxonomy based on EEQAs (effectiveness, extendibility, flexibility, functionality, reusability, and understandability) is critical for software development. It has several advantages for developers, including a structured approach to improving the quality of software systems. EEQAs have a significant impact on how users perceive and use software systems.

The effectiveness aspect ensures that the software system meets the users’ expectations. The proposed taxonomy of refactoring approaches aims to refine approaches, including EF, EC, ESb, ESP, and IPO, and improve the effectiveness of the systems. Using these approaches, the developers can fulfill the user’s requirements. In the same context, the functionality aspect is a user’s capacity to execute the necessary functions effectively. The proposed refactoring taxonomy exhibits many ways to apply refactoring, including EF, EC, ESb, ESP, IPO, PUF, and PDM, for enhancing the functionality of the software systems. By focusing on the refactoring approaches that strengthen effectiveness and functionality, the proposed refactoring taxonomy assists developers in prioritizing user satisfaction when making decisions.

Flexibility and extensibility are essential dimensions of software systems, which can affect how these systems are changed and adapted for future improvements and evolutions. Relatively to the proposed refactoring taxonomy, developers can select existing refactoring approaches that improve flexibility, including EF, EC, ESb, and ESP. In addition, the ESP refactoring approach can be used to improve extensibility. These approaches automate the coding refactoring to provide the developers with more modular, maintainable, and flexible software designs as requirements change. As a result, the effort and cost of the modifications will be diminished in the future.

The reusability of software systems is vital during the design and development process because it complements the software developers by enabling them to reuse existing code, libraries, and packages in other software programs or modules. In this context, the proposed refactoring taxonomy helps the developers choose suitable approaches: EF, EC, ESb, ESP, IPO, PUF, or PDM, which provide the packages, libraries, and shared components. By choosing these approaches, the developers can improve productivity and save time on development through code reusability. Thus, the code becomes more maintainable.

The understandability factor of software systems emphasizes the end users’ capacity to interact and use the systems efficiently and the developer’s capacity to implement and maintain them across various platforms without much trouble. The proposed refactoring taxonomy is the basis for the software developers to choose between the ESP or PUM refactoring approaches used to improve the understandability of the systems by making them more readable, modular, and documented. These refactoring approaches can be used to develop better readability, assist in maintenance and debugging, and decrease system complexity.

The proposed refactoring taxonomy that is developed can guide developers in making the selection of the appropriate refactoring approaches that help them to monitor and assess the effect of these approaches on EEQAs, with the support of the criteria for evaluating qualities like reusability, effectiveness, and extendibility, and the identification of precise measurement techniques. EEQAs, including extendibility and flexibility, play a significant role in dictating the software architecture design of software systems.

Sustainability in software engineering refers to the ability of systems to remain maintainable, adaptable, and reliable over time, while minimizing technical debt and supporting long-term evolution. The proposed taxonomy contributes to sustainability by providing a structured approach for selecting refactoring techniques that not only improve immediate code quality but also enhance maintainability, reduce future maintenance effort, and extend the lifecycle of software systems. By systematically applying appropriate refactoring practices, developers can ensure that systems remain adaptable to changing requirements, easier to test, and more resilient to faults, thereby supporting long-term software sustainability.

Additionally, the outcome of the proposed taxonomy can significantly contribute to the sustainability of software projects by promoting long-term viability and continuous maintenance. This taxonomy is built around the model of various EEQAs that are considered by software engineers, such as effectiveness, extendibility, flexibility, functionality, reusability, and understandability. Therefore, software developers can systematize quality enhancement procedures and match user expectations. The findings of the proposed taxonomy eliminate the possibility of the software systems being outdated and becoming ineffective in evolving user needs and new technologies, hence supporting the sustainability of the project.

In addition, by supporting potential advancements and implementing reusability, the outcome of taxonomy gives developers an opportunity to build new functionalities or enhance the existing ones on the already established codebase, decreasing the redundancy and the development time needed. Furthermore, this process is a key factor in the productivity of the software development team and ensures that the software remains sustainable, relevant, and useful in the long run.

Besides, the proposed taxonomy is designed to support system understanding and maintenance; as a result, developers can properly manage the software throughout its lifecycle. By ensuring understandable improvements in the quality attributes of the project and guiding developers with their architectural design decisions, the outcome of the taxonomy leads developers to informed choices that will improve the sustainability of the project as a whole.

The proposed refactoring taxonomy enables the designers to choose the best refactoring approaches that can enhance the EEQAs. Consequently, improving the EEQAs leads to making the software systems sustainable. In other words, this proposed refactoring taxonomy emphasizes these attributes, enhancing sustainability, evolution, and scalability by helping design software architecture that will continually adapt to changes and remain robust.

Overall, these outcomes demonstrate how adopting a structured approach to prioritizing the critical quality attributes and making the right choice on the software development lifecycle decisions has a strong effect on the sustainability of the software projects, as they will ensure the systems continue to meet the emerging application requirements.

## 5. Threats to validity

This part examines various types of validity threats and how to address them to increase confidence in the study’s outcomes. When conducting experiments, it is important to consider threats to validity, such as construct validity, conclusion validity, internal validity, and external validity.

The validity of the construction is a crucial concern because it is the theoretical backbone of any study, determining how well the theory matches the measurement scales or tools it uses. The ten most popular refactoring approaches used in practice by software practitioners were selected for this study to avoid subjectivity and bias in selection. In addition, this research utilized QMOOD as a reliable quality model to evaluate the effects of refactoring approaches on EEQAs.

The conclusion’s validity pertains to other researchers’ reproducibility of the study findings, and if they follow the exact procedure, they will obtain similar results. Forty-one independent experiments occurred through five case studies while building this taxonomy. Thus, the study’s findings are adequate to conclude. Furthermore, the experiments were conducted following a rigorous methodology to replicate the five case studies, and guidelines for implementing refactoring approaches were established.

The internal validity of a study is the extent to which confidence can be established between the treatment and the outcome. The five case studies selected for investigation and evaluation primarily focus on software refactoring. This indicates that they are reliable samples of the population. The analyzed case studies underwent no treatment other than refactoring for the sole observation of the impact of refactoring approaches across case studies. All case studies were treated identically under comparable circumstances. The states of the case studies post-refactoring were meticulously preserved to calculate the metrics and EEQAs. The experiments began with small-scale case studies and progressed to larger-scale case studies, enabling the researcher to acquire experience as the experiment advanced.

External validity is the extent to which the results can be applied in a larger context. It represents the possibility of generalizing the results. This study raised external validity; for instance, experiments were run on various case studies belonging to different domains of application and came in multiple sizes. This research examines the refactoring approaches used in Java projects primarily designed for Java systems. Java is recognized as one of the industry’s and academia’s most popular programming languages. The results cannot be guaranteed to be applied to other programming languages with varying refactoring approaches and tool support. Researchers are encouraged to expand this study to include other programming languages like JavaScript and Python

## 6. Conclusions

This paper addresses developers’ significant challenges in identifying suitable refactoring approaches to enhance software quality and facilitate maintenance tasks. By proposing a novel taxonomy based on estimated external quality attributes (EEQAs), this study provides developers with a systematic approach to effectively selecting and implementing refactoring approaches. The development of the proposed refactoring taxonomy involved three phases: an exploratory study, an experimental study, and a multi-case analysis. In the exploratory study, the ten most used refactoring approaches (Encapsulate Field, Extract Class, Extract Subclass, Extract Superclass, Hide Method, Introduce Parameter Object, Push Down Field, Pull Up Field, Push Down Method, and Pull Up Method), six estimated external quality attributes (effectiveness, extendibility, flexibility, functionality, reusability, and understandability), and five case studies (jEdit, jHotDraw, BMS, LMS, and PMS) were selected as the basis for taxonomy development. Subsequently, 41 experiments were carried out across the five case studies to evaluate the influence of refactoring approaches on EEQAs, culminating in the proposed refactoring taxonomy.

We developed the proposed refactoring taxonomy, categorizing refactoring into positive, negative, and ineffective classifications according to their influence on the EEQAs. To the software developers, the proposed refactoring taxonomy brings many benefits. It gives a structured framework for comprehension and classification of refactoring approaches based on their impact on EEQAs. Moreover, the proposed taxonomy enables practitioners to lead a uniform and standard refactoring practice, which supports collaboration and knowledge sharing among developers. Developer utilization of the proposed taxonomy can result in refactoring efforts aligning with critical qualities of EEQAs, such as effectiveness, extensibility, flexibility, functionality, reusability, and understandability. This, in turn, will lead developers to improve software quality, support future enhancements, promote reusability, facilitate system understanding and maintenance, and increase stakeholder satisfaction. Consequently, by emphasizing these EEQAs attributes, the proposed refactoring taxonomy enhances software sustainability by helping design architecture that continually adapts to changes and remains robust.

The proposed taxonomy offers software practitioners multiple bases for selecting the refactoring approaches according to which software properties they desire to improve or maintain. The proposed taxonomy is effective because it allows practitioners to visualize effective processes that are either desired characteristics of the industry or are neutral, but also recognize other processes that result in a negative outcome.

The proposed taxonomy allows resource utilization by determining the practices expected to be very effective, approaches with just a little impact, or practices with some tradeoff options. Such allows a feasible choice of effective tools for a certain type of software, as it is especially important for software practitioners. The proposed taxonomy realizes the importance of different refactoring approaches to EEQAs and allows a better understanding of each approach’s impact. The acquisition of such knowledge allows expert developers to develop a holistic understanding of various software design principles and how each refactoring influences the entire scope of changes.

The proposed taxonomy for classifying refactoring approaches represents significant advantages for software practitioners in the industry. It supplies functional help and guidance in the process of selecting and introducing the necessary refactoring approaches that correspond to particular EEQAs. The proposed taxonomy was built as a governance template in its organization and completeness, with classification according to the effects of refactoring approaches.

The proposed taxonomy can help software practitioners with the prescribed and applied refactoring approaches, which can assist in increasing the quality of software systems. The software complexity can be lowered, resulting in no quality loss throughout the process. The proposed taxonomy provides practitioners with the capacity to make more rational decisions that are proper to their goal because it enables them to map different refactoring approaches to EEQAs based on their impact on software systems. In other words, it eventually results in enhanced products. In conclusion, the proposed taxonomy is a secure tool for the software engineering field and offers a foundation for the next generation of refactoring practices.

## 7. Limitations and future directions

This study has certain limitations that provide opportunities for future exploration. First, while the taxonomy provides a structured classification of refactoring approaches based on estimated external quality attributes (EEQAs), its applicability in large-scale industrial systems has yet to be fully tested.

Different avenues can be explored in the future to advance the proposed refactoring taxonomy. Firstly, additional case studies can be carried out to validate the taxonomy across a broader range of software projects and environments. Longitudinal experiments that validate the effectiveness of the proposed taxonomy through real-world software development projects would provide valuable insights. Secondly, the proposed taxonomy could be further improved by introducing more refactoring approaches and EEQAs to give the developers a more versatile and flexible framework. Moreover, it could be possible for future research to examine the incorporation of automatic tool support for developers to apply the proposed taxonomy in their refactoring process successfully.

Additionally, integrating artificial intelligence (AI) and machine learning techniques could enable automated refactoring decisions guided by the taxonomy, thereby improving efficiency and reducing manual effort. Further exploration of sustainability aspects, such as energy efficiency and resource optimization, would also strengthen the taxonomy’s alignment with sustainable software engineering practices.

## References

[1] Traini L, Di Pompeo D, Tucci M, Lin B, Scalabrino S, Bavota G, et al. How Software Refactoring Impacts Execution Time. ACM Trans Softw Eng Methodol. 2021;31(2):1–23. doi: 10.1145/3485136

[2] McGuire S, Schultz E, Ayoola B, Ralph P. Sustainability is Stratified: Toward a Better Theory of Sustainable Software Engineering. In: 2023 IEEE/ACM 45th International Conference on Software Engineering (ICSE), 2023. 1996–2008. doi: 10.1109/icse48619.2023.00169

[3] Becker C, Chitchyan R, Duboc L, Easterbrook S, Penzenstadler B, Seyff N, et al. Sustainability Design and Software: The Karlskrona Manifesto. In: 2015 IEEE/ACM 37th IEEE International Conference on Software Engineering, 2015. doi: 10.1109/icse.2015.179

[4] Penzenstadler B, Femmer H. A generic model for sustainability with process- and product-specific instances. In: Proceedings of the 2013 workshop on Green in/by software engineering, 2013. 3–8. doi: 10.1145/2451605.2451609

[5] Venters CC, Lau L, Griffiths MK, Holmes V, Ward RR, Jay C, et al. The Blind Men and the Elephant: Towards an Empirical Evaluation Framework for Software Sustainability. Journal of Open Research Software. 2014;2(1). doi: 10.5334/jors.ao

[6] Khomh F, Penta MD, Guéhéneuc Y-G, Antoniol G. An exploratory study of the impact of antipatterns on class change- and fault-proneness. Empir Software Eng. 2011;17(3):243–75. doi: 10.1007/s10664-011-9171-y

[7] Moser R, Abrahamsson P, Pedrycz W, Sillitti A, Succi G. A Case Study on the Impact of Refactoring on Quality and Productivity in an Agile Team. Lecture Notes in Computer Science. Springer Berlin Heidelberg. 2008. p. 252–66. doi: 10.1007/978-3-540-85279-7_20

[8] Bavota G, De Lucia A, Marcus A, Oliveto R. Recommending Refactoring Operations in Large Software Systems. Recommendation Systems in Software Engineering. Springer Berlin Heidelberg. 2013. p. 387–419. doi: 10.1007/978-3-642-45135-5_15

[9] Fowler M, Beck K, Brant J, Opdyke W, Roberts D. Refactoring: Improving the Design of Existing Code. Addison-Wesley Professional. 2002.

[10] Fowler M, Beck K. Refactoring: Improving the Design of Existing Code. 2nd ed. Addison-Wesley Professional. 2019.

[11] Almogahed A, Mahdin H, Omar M, Zakaria NH, Muhammad G, Ali Z. Optimized Refactoring Mechanisms to Improve Quality Characteristics in Object-Oriented Systems. IEEE Access. 2023;11:99143–58. doi: 10.1109/access.2023.3313186

[12] Alomar EA, Wang T, Raut V, Mkaouer MW, Newman C, Ouni A. Refactoring for reuse: an empirical study. Innovations Syst Softw Eng. 2022;18(1):105–35. doi: 10.1007/s11334-021-00422-6

[13] Mumtaz H, Singh P, Blincoe K. Identifying refactoring opportunities for large packages by analyzing maintainability characteristics in Java OSS. Journal of Systems and Software. 2023;202:111717. doi: 10.1016/j.jss.2023.111717

[14] Abid C, Alizadeh V, Kessentini M, Ferreira N, Dig D. 30 Years of Software Refactoring Research: A Systematic Literature Review. arXiv preprint. 2020. https://arxiv.org/ftp/arxiv/papers/2007/2007.02194.pdf

[15] Almogahed A, Mahdin H, Omar M, Zakaria NH, Mostafa SA, AlQahtani SA, et al. A Refactoring Classification Framework for Efficient Software Maintenance. IEEE Access. 2023;11:78904–17. doi: 10.1109/access.2023.3298678

[16] Zhao Y, Yang Y, Zhou Y, Ding Z. DEPICTER: A Design-Principle Guided and Heuristic-Rule Constrained Software Refactoring Approach. IEEE Trans Rel. 2022;71(2):698–715. doi: 10.1109/tr.2022.3159851

[17] Nyamawe AS, Liu H, Niu Z, Wang W, Niu N. Recommending Refactoring Solutions Based on Traceability and Code Metrics. IEEE Access. 2018;6:49460–75. doi: 10.1109/access.2018.2868990

[18] Al Dallal J, Abdin A. Empirical Evaluation of the Impact of Object-Oriented Code Refactoring on Quality Attributes: A Systematic Literature Review. IIEEE Trans Software Eng. 2018;44(1):44–69. doi: 10.1109/tse.2017.2658573

[19] Kaur S, Singh P. How does object-oriented code refactoring influence software quality? Research landscape and challenges. Journal of Systems and Software. 2019;157:110394. doi: 10.1016/j.jss.2019.110394

[20] Lacerda G, Petrillo F, Pimenta M, Guéhéneuc YG. Code smells and refactoring: A tertiary systematic review of challenges and observations. Journal of Systems and Software. 2020;167:110610. doi: 10.1016/j.jss.2020.110610

[21] Fernandes E, Chávez A, Garcia A, Ferreira I, Cedrim D, Sousa L, et al. Refactoring effect on internal quality attributes: What haven’t they told you yet?. Information and Software Technology. 2020;126:106347. doi: 10.1016/j.infsof.2020.106347

[22] Almogahed A, Omar M, Zakaria NH. Recent Studies on the Effects of Refactoring in Software Quality: Challenges and Open Issues. In: 2022 2nd International Conference on Emerging Smart Technologies and Applications (eSmarTA), 2022. 1–7. doi: 10.1109/esmarta56775.2022.9935361

[23] Almogahed A, Omar M, Zakaria NH, Muhammad G, AlQahtani SA. Revisiting Scenarios of Using Refactoring Techniques to Improve Software Systems Quality. IEEE Access. 2023;11:28800–19. doi: 10.1109/access.2022.3218007

[24] Almogahed A, Mahdin H, Omar M, Zakaria NH, Alawadhi A, Barraood SO. Empirical Investigation of the Diverse Refactoring Effects on Software Quality: The Role of Refactoring Tools and Software Size. In: 2023 3rd International Conference on Emerging Smart Technologies and Applications (eSmarTA), 2023. 1–6. doi: 10.1109/esmarta59349.2023.10293407

[25] Alshayeb M. Empirical investigation of refactoring effect on software quality. Information and Software Technology. 2009;51(9):1319–26. doi: 10.1016/j.infsof.2009.04.002

[26] Rachatasumrit N, Kim M. An empirical investigation into the impact of refactoring on regression testing. In: 2012 28th IEEE International Conference on Software Maintenance (ICSM), 2012. 357–66. doi: 10.1109/icsm.2012.6405293

[27] Tsantalis N, Guana V, Stroulia E, Hindle A. A multidimensional empirical study on refactoring activity. In: 2013. 132–46. https://dl.acm.org/doi/10.5555/2555523.2555539

[28] Naiya N, Counsell S, Hall T. The Relationship between Depth of Inheritance and Refactoring: An Empirical Study of Eclipse Releases. In: 2015 41st Euromicro Conference on Software Engineering and Advanced Applications, 2015. 88–91. doi: 10.1109/seaa.2015.42

[29] Palomba F, Zaidman A, Oliveto R, De Lucia A. An Exploratory Study on the Relationship between Changes and Refactoring. In: 2017 IEEE/ACM 25th International Conference on Program Comprehension (ICPC), 2017. doi: 10.1109/icpc.2017.38

[30] Kannangara SH, Wijayanayake WMJI. An empirical exploration of refactoring effect on software quality using external quality factors. Int J on Adv in ICT for Emerging Countries. 2014;7(2):36. doi: 10.4038/icter.v7i2.7176

[31] Stroggylos K, Spinellis D. Refactoring--Does It Improve Software Quality?. In: Fifth International Workshop on Software Quality (WoSQ’07: ICSE Workshops 2007), 2007. 10–10. doi: 10.1109/wosq.2007.11

[32] Bavota G, De Lucia A, Di Penta M, Oliveto R, Palomba F. An experimental investigation on the innate relationship between quality and refactoring. Journal of Systems and Software. 2015;107:1–14. doi: 10.1016/j.jss.2015.05.024

[33] Soetens QD, Demeyer S. Studying the Effect of Refactorings: A Complexity Metrics Perspective. In: 2010 Seventh International Conference on the Quality of Information and Communications Technology, 2010. 313–8. doi: 10.1109/quatic.2010.58

[34] Wilking D, Khan UF, Kowalewski S. An Empirical Evaluation of Refactoring. E-Informatica Software Engineering Journal. 2007;1(1).

[35] Elish KO, Alshayeb M. Investigating the Effect of Refactoring on Software Testing Effort. In: 2009 16th Asia-Pacific Software Engineering Conference, 2009. 29–34. doi: 10.1109/apsec.2009.14

[36] Alshayeb M. Refactoring Effect on Cohesion Metrics. In: 2009 International Conference on Computing, Engineering and Information, 2009. 3–7. doi: 10.1109/icc.2009.12

[37] Halim A, Mursanto P. Refactoring rules effect of class cohesion on high-level design. In: 2013 International Conference on Information Technology and Electrical Engineering (ICITEE), 2013. 197–202. doi: 10.1109/iciteed.2013.6676238

[38] Almogahed A, Mahdin H, Omar M, Zakaria NH, Gu YH, Al-Masni MA, et al. A refactoring categorization model for software quality improvement. PLoS One. 2023;18(11):e0293742. doi: 10.1371/journal.pone.0293742 37917752 PMC10621946

[39] Chaparro O, Bavota G, Marcus A, Penta MD. On the Impact of Refactoring Operations on Code Quality Metrics. In: 2014 IEEE International Conference on Software Maintenance and Evolution, 2014. 456–60. doi: 10.1109/icsme.2014.73

[40] Abid C, Rzig DE, Ferreira T do N, Kessentini M, Sharma T. X-SBR: On the Use of the History of Refactorings for Explainable Search-Based Refactoring and Intelligent Change Operators. IIEEE Trans Software Eng. 2022;48(10):3753–70. doi: 10.1109/tse.2021.3105037

[41] Nyamawe AS. Mining commit messages to enhance software refactorings recommendation: A machine learning approach. Machine Learning with Applications. 2022;9:100316. doi: 10.1016/j.mlwa.2022.100316

[42] Almogahed A, Omar M, Zakaria NH. Refactoring Codes to Improve Software Security Requirements. Procedia Computer Science. 2022;204:108–15. doi: 10.1016/j.procs.2022.08.013

[43] Soares E, Ribeiro M, Gheyi R, Amaral G, Santos A. Refactoring Test Smells With JUnit 5: Why Should Developers Keep Up-to-Date?. IIEEE Trans Software Eng. 2023;49(3):1152–70. doi: 10.1109/tse.2022.3172654

[44] Kaya M, Conley S, Othman ZS, Varol A. Effective software refactoring process. In: 2018 6th International Symposium on Digital Forensic and Security (ISDFS), 2018. 1–6. doi: 10.1109/isdfs.2018.8355350

[45] Alizadeh V, Kessentini M, Mkaouer W, Ocinneide M, Ouni A, Cai Y. Interactive and Dynamic Multi-Objective Software Refactoring Recommendations. In: Proceedings of the 33rd ACM/IEEE International Conference on Automated Software, 2019. 1–30. https://deepblue.lib.umich.edu/bitstream/handle/2027.42/147343/papertse.pdf?sequence=1

[46] Alizadeh V, Ouali MA, Kessentini M, Chater M. RefBot: Intelligent Software Refactoring Bot. In: 34th IEEE/ACM International Conference on Automated Software Engineering (ASE), 2019. 823–34. https://ieeexplore.ieee.org/document/8952287

[47] Sousa L, Cedrim D, Garcia A, Oizumi W, Bibiano AC, Oliveira D, et al. Characterizing and Identifying Composite Refactorings. In: Proceedings of the 17th International Conference on Mining Software Repositories, 2020. 186–97. doi: 10.1145/3379597.3387477

[48] Almogahed MO, Zakaria NH. Categorization refactoring techniques based on their effect on software quality attributes. Int J Innov Technol Explor Eng. 2019;8(8S):439–45.

[49] Shahjahan A, haider Butt W, Ahmad AZ. Impact of refactoring on code quality by using graph theory: An empirical evaluation. In: 2015 SAI Intelligent Systems Conference (IntelliSys), 2015. 595–600. doi: 10.1109/intellisys.2015.7361201

[50] Kaur A, Kaur M. Analysis of Code Refactoring Impact on Software Quality. MATEC Web of Conferences. 2016;57:02012. doi: 10.1051/matecconf/20165702012

[51] Khrishe Y, Alshayeb M. An empirical study on the effect of the order of applying software refactoring. In: 2016 7th International Conference on Computer Science and Information Technology (CSIT), 2016. doi: 10.1109/csit.2016.7549471

[52] Fontana FA, Roveda R, Vittori S, Metelli A, Saldarini S, Mazzei F. On evaluating the impact of the refactoring of architectural problems on software quality. In: Proceedings of the Scientific Workshop Proceedings of XP2016, 2016. 1–8. doi: 10.1145/2962695.2962716

[53] Kádár I, Hegedűs P, Ferenc R, Gyimóthy T. A Manually Validated Code Refactoring Dataset and Its Assessment Regarding Software Maintainability. In: Proceedings of the The 12th International Conference on Predictive Models and Data Analytics in Software Engineering, 2016. 1–4. doi: 10.1145/2972958.2972962

[54] Kaur G, Singh B. Improving the quality of software by refactoring. In: 2017 International Conference on Intelligent Computing and Control Systems (ICICCS), 2017. 185–91. doi: 10.1109/iccons.2017.8250707

[55] Bashir RS, Lee SP, Yung CC, Alam KA, Ahmad RW. A Methodology for Impact Evaluation of Refactoring on External Quality Attributes of a Software Design. In: 2017 International Conference on Frontiers of Information Technology (FIT), 2017. 183–8. doi: 10.1109/fit.2017.00040

[56] Chávez A, Ferreira I, Fernandes E, Cedrim D, Garcia A. How does refactoring affect internal quality attributes?. In: Proceedings of the XXXI Brazilian Symposium on Software Engineering, 2017. 74–83. doi: 10.1145/3131151.3131171

[57] Mourad B, Badri L, Hachemane O, Ouellet A. Exploring the Impact of Clone Refactoring on Test Code Size in Object-Oriented Software. In: 2017 16th IEEE International Conference on Machine Learning and Applications (ICMLA), 2017. 586–92. doi: 10.1109/icmla.2017.00098

[58] Mumtaz H, Alshayeb M, Mahmood S, Niazi M. An empirical study to improve software security through the application of code refactoring. Information and Software Technology. 2018;96:112–25. doi: 10.1016/j.infsof.2017.11.010

[59] Alazzam I, Abuata B, Mhediat G. Impact of Refactoring on OO Metrics: A Study on the Extract Class, Extract Superclass, Encapsulate Field and Pull up Method. IJMLC. 2020;10(1):158–63. doi: 10.18178/ijmlc.2020.10.1.913

[60] Du. Bois T, Mens T. Describing the impact of refactoring on internal program quality. In: 2003. http://plg2.math.uwaterloo.ca/~migod/papers/2003/ELISAproceedings.pdf#page=39

[61] Du Bois B, Demeyer S, Verelst J. Refactoring - improving coupling and cohesion of existing code. In: 11th Working Conference on Reverse Engineering. doi: 10.1109/wcre.2004.33

[62] Elish KO, Alshayeb M. A Classification of Refactoring Methods Based on Software Quality Attributes. Arab J Sci Eng. 2011;36(7):1253–67. doi: 10.1007/s13369-011-0117-x

[63] Alshayeb M, Al‐Jamimi H, Elish MO. Empirical taxonomy of refactoring methods for aspect‐oriented programming. J Software Evolu Process. 2011;25(1):1–25. doi: 10.1002/smr.544

[64] Elish KO, Alshayeb M. Using Software Quality Attributes to Classify Refactoring to Patterns. JSW. 2012;7(2). doi: 10.4304/jsw.7.2.408-419

[65] Bhattacharyya S, Hassanien AE, Gupta D, Khanna A, Pan I. International Conference on Innovative Computing and Communications. Springer Singapore. 2019. doi: 10.1007/978-981-13-2354-6

[66] Malhotra R, Chug A. An empirical study to assess the effects of refactoring on software maintainability. In: 2016 International Conference on Advances in Computing, Communications and Informatics (ICACCI), 2016. 110–7. doi: 10.1109/icacci.2016.7732033

[67] Al Dallal J. Identifying refactoring opportunities in object-oriented code: A systematic literature review. Information and Software Technology. 2015;58:231–49. doi: 10.1016/j.infsof.2014.08.002

[68] Mariani T, Vergilio SR. A systematic review on search-based refactoring. Information and Software Technology. 2017;83:14–34. doi: 10.1016/j.infsof.2016.11.009

[69] Et.al AA. Empirical Studies on Software Refactoring Techniques in the Industrial Setting. TURCOMAT. 2021;12(3):1705–16. doi: 10.17762/turcomat.v12i3.995

[70] Almogahed A, Omar M, Zakaria NH, Alawadhi A. Software Security Measurements: A Survey. In: 2022 International Conference on Intelligent Technology, System and Service for Internet of Everything (ITSS-IoE), 2022. 1–6. doi: 10.1109/itss-ioe56359.2022.9990968

[71] Almogahed MO, Zakaria NH. Impact of software refactoring on software quality in the industrial environment: A review of empirical studies. In: Proceedings of Knowledge Management International Conference (KMICe), Sarawak, Malaysia, 2018. 229–34. http://soc.uum.edu.my/kmice/proceedings/proc/2018/pdf/CR61.pdf

[72] Kim M, Zimmermann T, Nagappan N. An Empirical Study of RefactoringChallenges and Benefits at Microsoft. IIEEE Trans Software Eng. 2014;40(7):633–49. doi: 10.1109/tse.2014.2318734

[73] Gatrell M, Counsell S. The effect of refactoring on change and fault-proneness in commercial C# software. Science of Computer Programming. 2015;102:44–56. doi: 10.1016/j.scico.2014.12.002

[74] Ouni M, Kessentini H, Sahraoui M, Ó Cinnéide K, Deb K, Inoue K. A Multi-Objective Refactoring Approach to Introduce Design Patterns and Fix Anti-Patterns. In: First North American Search Based Software Engineering Symposium, NASBASE, 2015. 1–15. https://kir.ics.es.osaka-u.ac.jp/lab-db/betuzuri/archive/990/990.pdf

[75] Almogahed A, Omar M. Refactoring Techniques for Improving Software Quality: Practitioners’ Perspectives. Journal of Information and Communication Technology. 2021;20. doi: 10.32890/jict2021.20.4.3

[76] Jabangwe R, Börstler J, Šmite D, Wohlin C. Empirical evidence on the link between object-oriented measures and external quality attributes: a systematic literature review. Empir Software Eng. 2014;20(3):640–93. doi: 10.1007/s10664-013-9291-7

[77] Bansiya J, Davis CG. A hierarchical model for object-oriented design quality assessment. IIEEE Trans Software Eng. 2002;28(1):4–17. doi: 10.1109/32.979986

[78] Pham V, Lokan C, Kasmarik K. A Better Set of Object-Oriented Design Metrics for Within-Project Defect Prediction. In: Proceedings of the Evaluation and Assessment in Software Engineering, 2020. 230–9. doi: 10.1145/3383219.3383243

[79] Goyal PK, Joshi G. QMOOD metric sets to assess quality of Java program. In: 2014 International Conference on Issues and Challenges in Intelligent Computing Techniques (ICICT), 2014. 520–33. doi: 10.1109/icicict.2014.6781337

[80] Couto CMS, Rocha H, Terra R. A Quality-oriented Approach to Recommend Move Method Refactorings. In: Proceedings of the XVII Brazilian Symposium on Software Quality, 2018. 11–20. doi: 10.1145/3275245.3275247

[81] Kaur A. A Systematic Literature Review on Empirical Analysis of the Relationship Between Code Smells and Software Quality Attributes. Arch Computat Methods Eng. 2019;27(4):1267–96. doi: 10.1007/s11831-019-09348-6

[82] Payroll Management System. [cited on 11 February 2021]. Available online: https://code-projects.org/library-management-system-in-java-with-source-code/

[83] Source Code & Projects. [cited on 18 August 2019]. Available online: https://code-projects.org/library-management-system-in-java-with-source-code/

[84] Banking System Management. [cited on 25 August 2020]. Available online: https://github.com/derickfelix/BankApplication

[85] jHotDraw Files. (cited on 25 July 2019). Available online: https://sourceforge.net/projects/jHotDraw/files/JHotDraw/5.2/

[86] jEdit Files. (cited on 25 November 2019)Available online. https://sourceforge.net/projects/jedit/files/jedit/5.5.0/

[87] JDeodorant. (cited on 02 September 2019). Available online: https://marketplace.eclipse.org/content/jdeodorant

[88] Metrics 3 - Eclipse Metrics Plugin Continued ‘Again.’ [accessed on 05 August 2019). Available online: https://github.com/qxo/eclipse-metrics-plugin

[89] Eclipse Foundation. [cited on 25 July 2019]. Available online: https://www.eclipse.org/downloads/

[90] Eisenhardt KM. Building Theories from Case Study Research. The Academy of Management Review. 1989;14(4):532. doi: 10.2307/258557

[91] Maglio PP, Lim C-H. Innovation and Big Data in Smart Service Systems. jim. 2016;4(1):11–21. doi: 10.24840/2183-0606_004.001_0003

[92] Ketokivi M, Choi T. Renaissance of case research as a scientific method. J of Ops Management. 2014;32(5):232–40. doi: 10.1016/j.jom.2014.03.004

[93] Lim C, Kim K-J, Maglio PP. Smart cities with big data: Reference models, challenges, and considerations. Cities. 2018;82:86–99. doi: 10.1016/j.cities.2018.04.011

[94] Almogahed A, Mahdin H, Hyeon Gu Y, Al-Masni MA, Alzaeemi SA, Omar M, et al. Multi-Classification Refactoring Framework Using Hopfield Neural Network for Sustainable Software Development. IEEE Access. 2025;13:31785–808. doi: 10.1109/access.2025.3542087

